# Clinical implementation of a 3D4K-exoscope (Orbeye) in microneurosurgery

**DOI:** 10.1007/s10143-021-01577-3

**Published:** 2021-06-18

**Authors:** Judith Rösler, Stefan Georgiev, Anna L. Roethe, Denny Chakkalakal, Güliz Acker, Nora F. Dengler, Vincent Prinz, Nils Hecht, Katharina Faust, Ulf Schneider, Simon Bayerl, Marcus Czabanka, Martin Misch, Julia Onken, Peter Vajkoczy, Thomas Picht

**Affiliations:** 1 Department of Neurosurgery, Charité - Universitätsmedizin Berlin, Charitéplatz 1, 10117 Berlin, Germany; 2 Berlin Simulations and Training Center (BeST), Charité-Universitätsmedizin Berlin, Berlin, Germany; 3 Cluster of Excellence: “Matters of Activity. Image Space Material”, Humboldt University, Berlin, Germany

**Keywords:** 3-Dimensional, Exoscope, Intraoperative visualization, Neurosurgery

## Abstract

Exoscopic surgery promises alleviation of physical strain, improved intraoperative visualization and facilitation of the clinical workflow. In this prospective observational study, we investigate the clinical usability of a novel 3D4K-exoscope in routine neurosurgical interventions. Questionnaires on the use of the exoscope were carried out. Exemplary cases were additionally video-documented. All participating neurosurgeons (n = 10) received initial device training. Changing to a conventional microscope was possible at all times. A linear mixed model was used to analyse the impact of time on the switchover rate. For further analysis, we dichotomized the surgeons in a frequent (n = 1) and an infrequent (n = 9) user group. A one-sample Wilcoxon signed rank test was used to evaluate, if the number of surgeries differed between the two groups. Thirty-nine operations were included. No intraoperative complications occurred. In 69.2% of the procedures, the surgeon switched to the conventional microscope. While during the first half of the study the conversion rate was 90%, it decreased to 52.6% in the second half (*p* = 0.003). The number of interventions between the frequent and the infrequent user group differed significantly (*p* = 0.007). Main reasons for switching to ocular-based surgery were impaired hand–eye coordination and poor depth perception. The exoscope investigated in this study can be easily integrated in established neurosurgical workflows. Surgical ergonomics improved compared to standard microsurgical setups. Excellent image quality and precise control of the camera added to overall user satisfaction. For experienced surgeons, the incentive to switch from ocular-based to exoscopic surgery greatly varies.

## Introduction

With the introduction of the surgical microscope in the 1960s, the spectrum and safety of neurosurgical interventions increased due to improved optical magnification and illumination of the surgical field. A disadvantage of this revolutionary technique is the physical strain when the optical system is positioned outside the physiological body axis. Particularly, persistent flexion of the cervical spine may lead to long-term effects on the musculoskeletal system and compromises the surgical performance [1]. Bending the head 30 degrees off the neutral position results in an up to four times increased load on the cervical spine, with consecutive loss of lordosis and compensational thoracic hyperkyphosis [2, 3]. Technical improvements like neuroendoscopes, which decouple the visual from the working axis, particularly in regard of operations at extreme angles, have demonstrated superior ergonomics during surgery [4]. However, while endoscopes offer a more flexible viewing while maintaining a physiological posture, the shorter focal range length causes reduced depth perception and a lack of stereoscopic vision [5, 6].

With the increased technical complexity of neurosurgical interventions, there is a growing demand for microscopic units with multimodal inputs and flexible manoeuvrability. Current limitations of the conventional microscope include the optical system’s low ability for augmenting the surgeon’s view with additional information. Furthermore, the bulky corpus of binocular microscopes reduces setup flexibility and hinders the integration of robotics into the operating theatre.

The new generation of high-resolution exoscopes claims to overcome those limitations by enabling a flexible and ergonomic working environment, combined with an excellent image quality and fully digital image processing. The aim of our study was to investigate the clinical implementation of a modern 3D4K system and to analyse its advantages and disadvantages as well as its influence on intraoperative ergonomics and workflows.

## Methods and materials

### Study design

A prospective case collection was performed to evaluate the exoscope Olympus Orbeye (Sony Olympus Medical Solutions, Tokyo, Japan) in a routine clinical setup for cranial, spinal and peripheral nerve procedures. Cases were documented within a period of 6 months in 2019 and 2020. Inclusion criteria contained age over 18 years. Exclusion criteria comprised emergency and high complexity interventions (category V (intraventricular lesions) and VI (cerebrovascular surgeries)) according to the Gonen classification [7].

After a general training and introduction, the use of the exoscope was left to each participating surgeon’s discretion. Changing to a conventional operative microscope was possible at all times.

Case documentation covered three categories. (1) Case information included age, histology, location, type of procedure, surgical approach, operating time, and intraoperative complications. Case complexity for intracranial lesions was classified using the Gonen categories [7], including category I for intracranial hematoma or abscess; II for intraaxial, non-eloquent areas or convexity meningioma; III for intraaxial, eloquent tumours and microvascular decompression of the trigeminal nerve; and IV for skull base pathologies or parasagittal meningioma with involvement of the venous sinus. (2) Utilization comprised the documentation of the intraoperative setup, use and positioning of the monitor and camera, viewing angles, handling of the foot pedal as well as the description of technical problems. The observer rated the surgical performance with regard to smoothness of surgical tasks, exposure of risk structures and tissue resection by noting motion hesitations through on-site documentation and subsequent assessment of the intraoperative video recording. (3) Questionnaires for the participating physicians included three parts. First, the intraoperative surgical satisfaction in terms of suitability, image quality and ergonomics was rated using a Likert scale from one to five. Secondly, the system usability scale (SUS) [8] evaluated the exoscope experience based on a scoring system from 1 (strongly disagree) to 5 (strongly agree). Thirdly, the task load index (TLX) [9] used a numerical rating scale, ranging from 0 (no impact) to 20 (highest impact), to assess the general surgical demand while utilizing the exoscope.

### Technical specifications

The Olympus Orbeye is a fully digital 3D4K extracorporeal telescope (exoscope), consisting of a main unit with a UNIX-based control console, a robotically assisted arm and a camera unit at the end of the robotic arm. The arm is repositioned either manually in X–Y-Z-axes or via a foot-controlled joystick horizontally. Buttons on the camera unit allow the control of basic functions like zoom and focus. The operative field is presented at a resolution of 3860 × 2160 pixels on a 3D 55″ and/or 33″ monitor with the help of passive polarized glasses (Sony Corporation, Tokyo, Japan). The magnification provided by the device is a 13-time optical zoom and two-time digital zoom, resulting in up to 26 times in total. Focal length ranges from 220 to 550 mm and the field of view from 7.5 to 171 mm. Infrared sensors for the use of ICG angiography and blue light for 5-ALA fluorescence are available. In addition to the joystick, the foot pedal provides 10 buttons for the control of the main functions of the device.

### Statistical analysis

To evaluate the existence of a potential learning effect on the switch rate to the standard microscope, we dichotomized the surgeon’s group into a frequent user (n = 1) and an infrequent group (n = 9) and divided the study interval in a first (n = 20 interventions) and a second half (n = 19). Determining the variable “time” as a fixed effect, the “number of performed cases” as a random effect and the “numbers of switchovers” as the depended variable, we performed a linear mixed model. The model was corrected for both surgeon cohorts performing interventions at all times of the study. To analyse weather there was a significant difference between the number of surgeries of frequent and infrequent exoscope users, we performed a one-sample Wilcoxon signed rank test. Statistical differences were significant if the p value was < 0.05.

As this study follows an explorative design, the remaining data analysis was executed based on descriptive statistics. Numeric parameters and the results of the surgeon’s questionnaires are presented as a median with its corresponding range and confidence interval (95% CI).

## Results

### Case characteristics

In total, 37 patients underwent 39 procedures, entirely or partially with the use of the exoscope. One patient had a second treatment for semi-elective haematoma evacuation of a delayed postoperative bleeding. Another patient initially received subdural plate electrode placement and consecutively surgery for epileptic focus resection.

The patient population included 62.2% women (n = 23) and 37.8% men (n = 14) with a median age of 55 years (range: 19–84). Cranial tumour surgeries varied from resections of superficial metastases (Gonen category II) to eloquent low-grade gliomas (Gonen III) as well as anterior clinoidal meningioma and suprasellar craniopharyngioma (Gonen IV) (Table 1). Epilepsy procedures included two temporal pole resections with hippocampectomy, three epileptical focus resections and one electrode placement procedure. The category “Brain–other” contained one haematoma evacuation and one trigeminal nerve decompression.

**Table 1:** Patient’s baseline criteria and histological diagnosis

Patient	Age	Sex	Pathology/diagnosis	Case complexity (Gonen)	Case complexity (subjective)
1	63	f	Glioblastoma WHO °IV	3	3
2	73	f	Metastasis (spinal)	n/a (not applicable)	3
3	65	f	Glioblastoma WHO °IV	4	4
4	55	m	Solitary fibrotic tumour (spinal)	n/a	3
5	50	f	Trigeminal neuralgia	3	3
6	47	f	Disc herniation (cervical)	n/a	3
7	19	m	Epilepsy	2	2
8	45	f	Neurinoma WHO °I (peripheral)	n/a	3
9	68	m	Pituitary adenoma WHO °I	4	2
10	54	f	Neurinoma WHO °I (thoracic)	n/a	3
11	70	f	Meningioma WHO °I	4	3
12	54	f	Craniopharyngeoma °I	4	5
13	58	f	Xanthoastrocytoma WHO °III	3	3
14	63	f	Glioma WHO °II	2	2
15	61	m	Glioblastoma WHO °IV	2	2
16	77	m	Diffuse glioma WHO °II	3	3
17	84	f	B-cell lymphoma (cranial)	2	3
18	57	m	Neurinoma WHO °I (cervical)	n/a	2
19	77	f	Metastasis (cranial)	3	3
20	39	m	Anaplastic Astrocytoma °II	3	3
21	22	f	Epilepsy	3	5
22	55	f	Glioblastoma WHO °IV	2	2
23	52	m	Spinal cord stenosis (lumbar)	n/a	4
24	26	f	Haemangioblastoma WHO °I (cranial)	3	3
25	69	m	Metastasis (cranial)	3	4
26	35	f	Metastasis (cranial)	2	4
27	26	f	Epilepsy	2	2
28	36	m	Epilepsy	2	2
29	34	f	Epilepsy	3	5
30	50	f	Spinal cord stenosis (lumbar)	n/a	2
31	48	f	Peripheral nerve	n/a	3
32	60	m	Glioblastoma WHO °IV	3	3
33	79	m	Disc herniation (lumbar)	n/a	4
34	28	f	Haemangioma (cranial)	4	4
35	31	m	Anaplastic glioma WHO °III	2	3
36	75	f	Disc herniation	n/a	1
27	26	f	Epilepsy	2	2
37	60	m	Neurofibroma WHO °I (peripheral)	n/a	3
3	65	f	Haematoma (cranial)	1	2

*n/a* Not applicable

The overall case complexity was perceived as low in 3.7% of the interventions, relatively low in 33.3%, intermediate in 37%, difficult in 22.2% and very difficult in 3.7% (Table 1).

Spinal surgery included microsurgical decompression for disc herniation or spinal stenosis, tumour resection and additional fusion procedures. The peripheral nerve procedures involved two femoral schwannoma resections and one neurolysis and decompression of the radial nerve.

The median surgical time was 112 min (range: 35–333 min). Out of eighteen brain tumour cases, 4 patients were diagnosed with glioblastoma WHO grade °IV and 5 with glioma grade °II or °III (Table 1).

Ten surgeons with 5 to 25 years of experience performed the interventions. One of them (10 years of experience) carried out nearly the half (41%) of all procedures with a significant higher number of interventions (*p* = 0.007) than the remaining participants. All procedures (30.8%) were performed exclusively with the use of the Orbeye, whereas the frequent operator completed 50% of the surgeries without conversion to the conventional microscope.

The conventional microscope was used in particular for the approach of deep (e.g. hippocampus) or vulnerable structures (e.g. cervical spinal cord, anterior clinoid region). During the observation period, the exclusive use of the exoscope increased with a conversion rate of 90% in the first (n = 20 interventions) and 52.6% in the second half (n = 19) of the study (*p* = 0.003).

No intraoperative complications occurred. Twenty-seven patients presented postoperatively with no change of their neurological condition, while nine patients improved. Overall, surgical time, resection rates and clinical outcomes were comparable to the department’s standard results of conventional binocular microsurgical procedures.

### Questionnaires

**Fig. 1:**
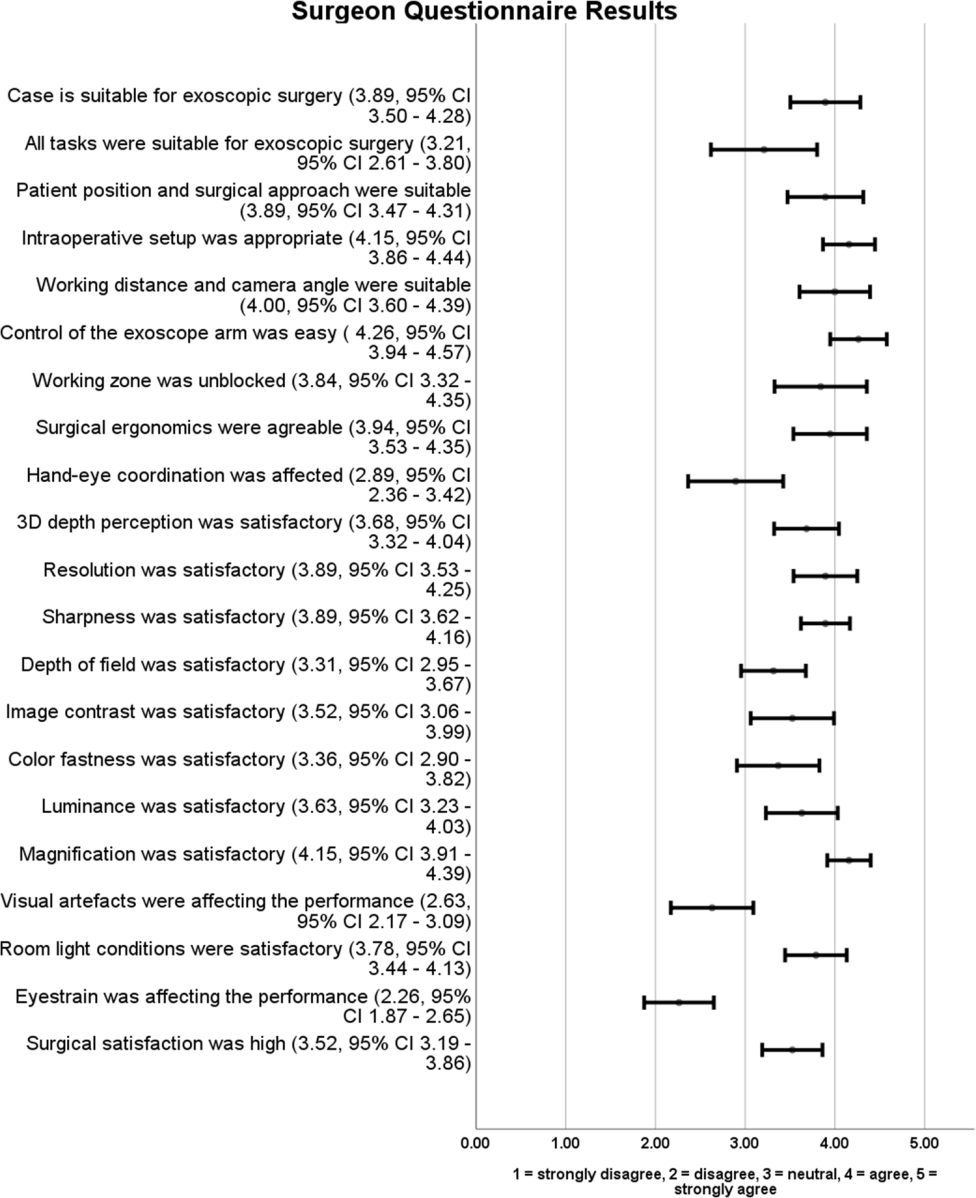
The four main positions of the Orbeye camera in relation to the surgeon's plane of view

The questionnaires were available for all participating surgeons and each procedure. In 73.7%, the surgeons postoperatively agreed that the case was suitable for exoscopic surgery. Most favourable categories were handling of the camera, intraoperative setup, magnification, resolution, working distance, camera angle and surgical ergonomics, closely followed by image sharpness as well as an unblocked working zone. A 3D depth perception, luminance, image contrast and depth of field were valued neutral to good. Impaired hand–eye coordination and visual artefacts were main reasons for surgical dissatisfaction. Eyestrain did not affect the general performance. Alike, vestibular symptoms caused by the 3D glasses did not occur. The overall surgical satisfaction ranged from neutral to high. The detailed results are presented in Fig. 1.

**Fig. 2:**
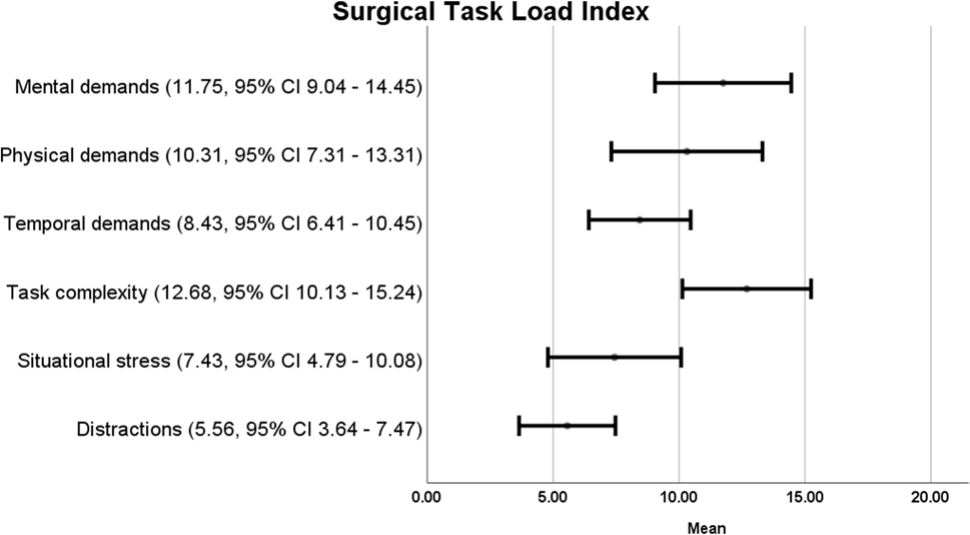
Results of the surgeon’s questionnaire (5-point Likert scale)

The surgical task load index is displayed in Fig. 2. The categories “cognitive and physical demands” settled in the median of the numerical rating scale. “Temporal demands” varied from low to medium impact on the surgical performance, whereas “situational stress” and “distraction” were rated as least disruptive.

**Fig. 3:**
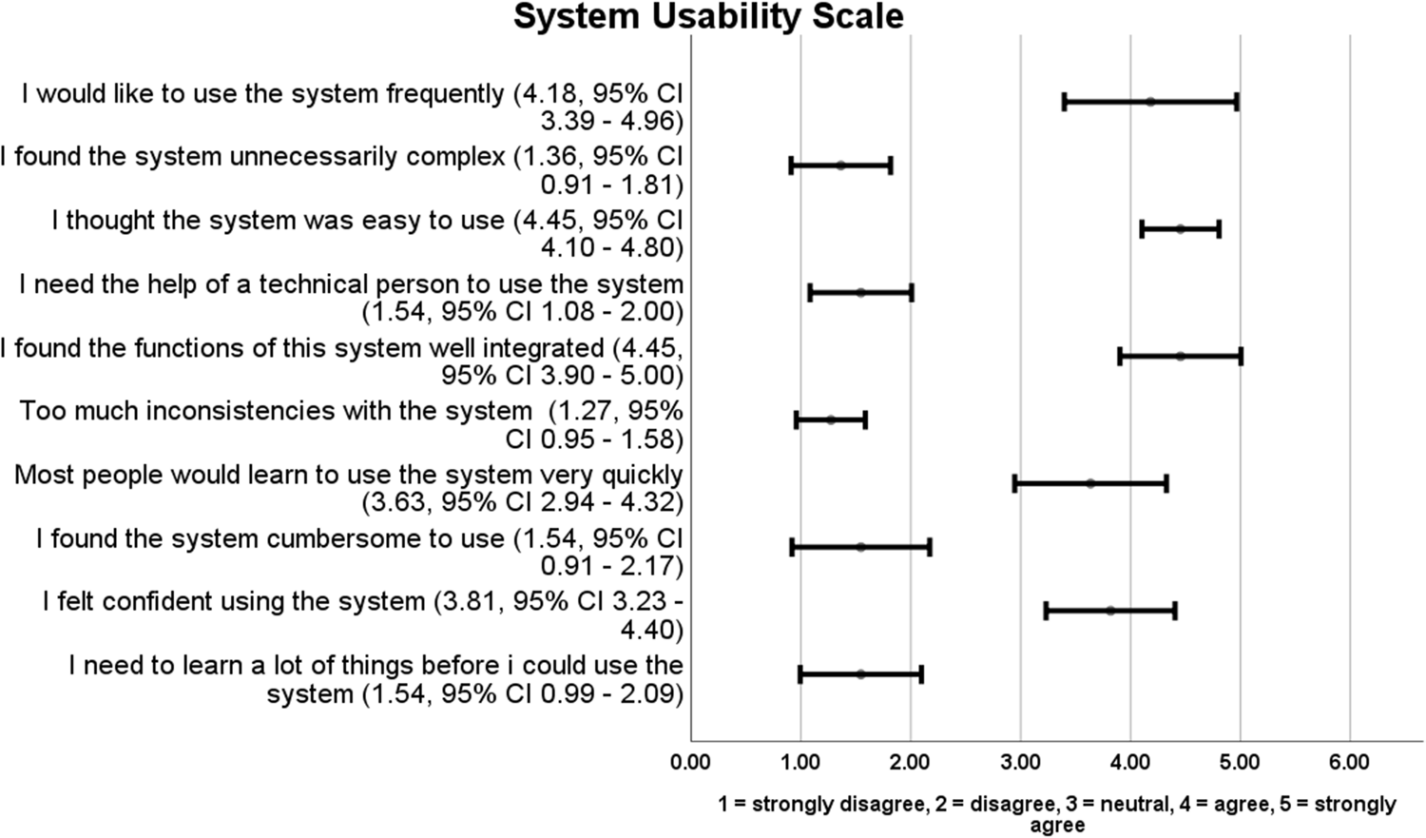
Results of the surgical task load index (visual analogue scale, ranging from 0 to 20)

In the system usability scale (Fig. 3), 58.9% of the surgeons agreed (12/39) or strongly agreed (11/39) on willingness to use the Orbeye more frequent. The exoscope was assessed as easy to handle, user-friendly and intuitive. The need for further technical support was low and the learning curve was rated as steep.

### Exoscope utilization

**Fig. 4:**
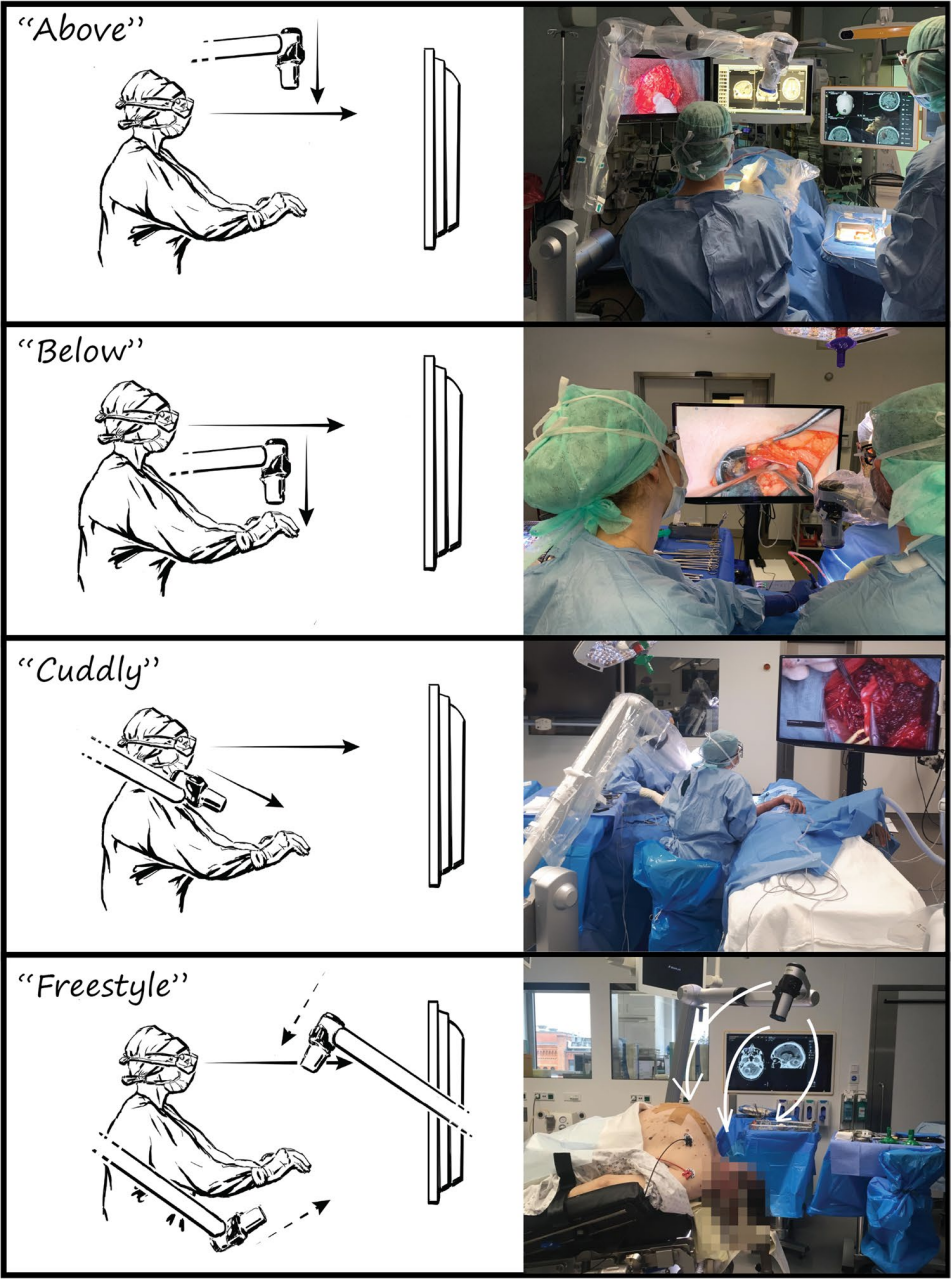
Results of the system usability scale (5-point Likert scale)

Documentation on the use of the exoscope included 10 additional cases with extensive video recording for post hoc evaluation. Depending on the positioning, the surgical approach and the patient’s specific anatomy, four main orientations of the Orbeye camera in relation to the surgeon's plane of view were observed (Fig. 4). For fronto-lateral, temporal or suboccipital approaches, the camera usually projected over the left shoulder of the surgeon, while the monitor was positioned at the bottom of the patient. For lumbar procedures, the monitor was placed opposite to the main surgeon. Finding the optimal exoscope position for peripheral nerve interventions remained challenging, as frequent changes of the camera orientation required subsequent monitor repositioning.

The 55-inch screen was used at a distance of 150 to 200 cm. The viewing angle varied between 0° and 10° in 90% of the cases. A positioning conflict with other devices occurred twice. In three cases, the screen orientation had to be readjusted during the procedure. In 3 of 6 procedures performed with an assistant, the integration of the assisting surgeon was hampered by an impaired view of the monitor, resulting in disturbed hand–eye coordination. The foot pedal was used in 7 cases (17.9%), 5 to 20 times per procedure. In one case, the foot pedal conflicted with other pedals for coagulation and drilling. In 4 cases (10.3%), the surgery was performed under dimmed light conditions. The main surgeon was sitting nearly half (49%) of the time. The head was upright in a neutral position in 86% of the time. Four times light reflections from the surgical head ring occurred, in particular when the camera did not point perpendicular onto the operative field. Overall, no technical problems were observed.

Camera repositioning was performed a median of 9 times (range: 3–19). The autofocus was used during 6 out of 10 video-recorded procedures. In the remaining 4 cases, manual focus adjustment was observed a median of 5 times (range: 3–11). In 8 out of 10 cases, movements were smooth during the entire surgical procedure while in 2 cases, hesitations as well as repetitive and compensatory movements were observed. Either of them was a radial nerve decompression with an intraoperative need for repositioning the camera multiple times (n = 19). The other operation was a transcranial resection of a craniopharyngioma with reduced stereopsis compared to ocular-based microscopy. Both surgeons had a low level of experience with exoscopic surgery with one carrying out 5.1% (2/39) of the procedures and the other 7.7% (3/39).

## Discussion

The exoscope in this study was assessed as an intuitive, flexible and ergonomic visualisation tool, providing high quality imagery, sufficient magnification and 3D depth perception [6, 10, 11]. Similar to former studies, complication rates were low and no negative impact on the patient’s outcome occurred [5, 10, 12]. In this series, the exoscope was feasible for a broad range of procedures with different complexities.

In our study, 30% of the surgeries were exclusively completed with the exoscope, while the frequent Orbeye user performed 50% of the interventions without ocular-based assistance. The infrequent exoscope surgeons tended to switch to the conventional microscope for approaches of critical and/or deep anatomical areas. Thus, during two anterior temporal lobectomies, the temporal pole resection was performed exoscope-guided, while the hippocampal structures were resected with the conventional microscope. Similarly, ocular-based microscopy was preferred for saphenous nerve graft transplantation with a 10–0 micro-sutures. The main reason for changing to conventional microscopy was a more familiar handling regarding hand–eye coordination and depth perception. The significant lower switchover to the microscope in the second half of the study (90% vs. 52.6%, *p* = 0.003) highlights the importance of device training.

Particularly in spine surgery, the default settings for contrast and depth of field were unsatisfying. For intracranial procedures, the pre-assigned settings for contrast occasionally caused overexposure of the white matter, as previously described [13]. Nevertheless, this limitation could be overcome by individual modification of the corresponding parameters. In our study, only one adjustment towards a broader depth of field was noted.

Procedures that require working from different positions around the operating table are a challenge for the setup. While the exoscope itself can be easily and flexibly repositioned, the position of the 55-inch monitor cannot be simply changed due to the limited space available. Careful planning of the setup is therefore necessary to achieve an optimal viewing angle and to avoid light reflections. A possible solution to this problem is the use of a smaller 33″ monitor mounted on the ceiling arm. In our preliminary experience, this allowed us to react flexibly to changes in the working axis while maintaining optimal viewing angles.

In our study, the optimal distance between the surgeon and the 55-inch screen was between 150 and 200 cm. The foot pedal was not used regularly, mainly due to personal preferences.

As described in other studies, the exoscope proved to be easy to use [6]. Control of the exoscopic arm, intraoperative ergonomics, image sharpness, resolution and magnification were rated positively [10]. The free working zone was also assessed positive. Ergonomics were perceived as favourable in comparison to the conventional microscope. Enabling an upright head position in 86% of the exoscopic operation time in our study was superior to a lately described upright head posture of 52% during ocular-based cranial surgeries [14].

While no neurovascular surgeries were performed in our study, other studies have shown those procedures feasible for exoscopic surgery as well [15]. Apart from the use in neurosurgery, ENT surgeons have reported success and high satisfaction [16, 17].

A limitation was the high number of different surgeons over a long observation period so that the number of cases per surgeon was relatively low and the learning curve consequently flat. Moreover, video recordings, which revealed valuable information on ergonomic aspects, the intraoperative workflow and positioning of technical devices, were only partly available.

In summary, exoscopic surgery is a viable tool for a variety of procedures, which provides high image quality with sufficient magnification, zoom and luminance. Switching to exoscopic surgery requires a variable time of adaptation. Particularly in the initial phase, accurate planning of the intraoperative setup and case-specific optimization of image settings are mandatory to ensure high compliance of the surgical team. Regular use of the exoscope is required to allow a smooth transition to exoscopic surgery and to facilitate adaptation. Successful and satisfactory usage of exoscopic surgery depends primarily on the surgeon's previous experience and his general comfort with stereoscopic imaging and screen-based surgery. The superiority of ergonomics compared to conventional ocular-based microscopy should be further evaluated.

## Data Availability

Not applicable.

## Code availability

All graphics and analysis were created with the aid of SPSS Version 24 (IBM Corporation, New York, USA).
